# Evaluating Large Language Models for AI-Assisted Decision Support in Legal Capacity Assessment: A Comparative Study Using Interdisciplinary Medical Board Recommendations as the Expert Medical Reference Standard

**DOI:** 10.3390/healthcare14152261

**Published:** 2026-07-24

**Authors:** Halit Canberk Aydogan, Muhammet Sevindik, Zeynep Unat Öztürk, Şükran Kaygısız, Hacer Yaşar Teke

**Affiliations:** 1 Department of Forensic Medicine, Ordu University Training and Research Hospital, Ordu 52200, Türkiye; zeynepunat@hotmail.com (Z.U.Ö.); hacer.hgulderen2004@gmail.com (H.Y.T.); 2 Department of Psychiatry, Ordu University Training and Research Hospital, Ordu 52200, Türkiye; muhammetsevindik_52@hotmail.com; 3 Department of Neurology, Ordu University Training and Research Hospital, Ordu 52200, Türkiye; skrnkygsz52@gmail.com

**Keywords:** large language models, guardianship, legal capacity, forensic decision support, artificial intelligence

## Abstract

**Background:** Legal capacity assessment requires multidisciplinary evaluation integrating cognitive, functional, neurological, psychiatric, and medico-legal information. Although large language models (LLMs) have shown promise in structured clinical reasoning, their role in supporting legal capacity assessment remains unclear. This study evaluated the performance of LLMs as AI-assisted decision-support tools using standardized medico-legal case vignettes, with interdisciplinary medical board (IMBD) recommendations under the Turkish Civil Code (TCC) as the expert medical reference standard; IMBD recommendations constitute an expert medical reference standard rather than final judicial determinations. **Methods:** We retrospectively analyzed 234 court-referred adult cases (2018–2024). Standardized, anonymized medico-legal case vignettes were independently evaluated by ChatGPT-5.2, Gemini 3 Pro, and Claude 4.5 Sonnet. Model outputs were compared with IMBD recommendations. The models were evaluated as AI-assisted decision-support tools and did not replace or influence clinical or judicial decision-making. Performance was assessed using accuracy, macro-F1, Cohen’s κ, AUC, calibration, decision-curve analysis, test–retest reliability, and human-factor outcomes; probability-based metrics were derived from secondary logistic models fitted to the categorical model outputs. **Results:** Article 405 was the most frequent outcome (65.8%). Dementia increased the likelihood of Article 405 recommendations (OR 6.5, 95% CI 1.2–35.2; *p* = 0.029), whereas higher Activities of Daily Living scores were protective (OR 0.96; *p* = 0.004). Gemini 3 Pro achieved the highest accuracy (86.3%), while ChatGPT-5.2 achieved the highest macro-F1 score (0.79) and AUC (0.91). Agreement with IMBD recommendations ranged from κ = 0.60 to 0.73, with high temporal stability (κ = 0.87–0.95); pairwise differences between the three models were not statistically significant after Holm correction. Performance was highest for cases in which Article 405 was recommended and for cases in which no guardianship was recommended, but remained limited for Article 408 (29.6% accuracy). The mean System Usability Scale score was 72.4, and safety flags were identified in 4 of 234 cases (1.7% of cases, corresponding to 4 of 702 individual model outputs). **Conclusions:** LLMs demonstrated agreement with IMBD recommendations on standardized medico-legal case vignettes, supporting further investigation of their potential role as AI-assisted decision-support tools under expert supervision. Because errors in this domain can directly affect fundamental rights, the use of AI in the legal system and in sensitive medical fields carries substantial risks and must remain strictly limited to expert-supervised decision support. Further prospective studies are needed to evaluate their safe integration into medico-legal practice.

## 1. Introduction

Legal capacity assessment is a high-stakes multidisciplinary medical evaluation that informs judicial decisions affecting personal autonomy, healthcare, financial management, and legal protection. Legal capacity constitutes a fundamental principle of civil law, enabling individuals to exercise rights, enter legally binding agreements, and make decisions concerning property, healthcare, and personal affairs [1,2,3]. Among legal interventions, guardianship and conservatorship represent the most restrictive measures, affecting more than 1.3 million adults in the United States and involving assets exceeding $50 billion as of 2018 [4,5]. In response to growing ethical concerns, Article 12 of the United Nations Convention on the Rights of Persons with Disabilities has promoted a shift toward supported decision-making models that seek to minimize disability-based restrictions on legal capacity while preserving individual autonomy [6,7].

Several jurisdictions, including Sweden, Germany, the United Kingdom, and Switzerland, have restructured guardianship frameworks to better preserve individual autonomy through more refined assessment processes [8,9,10,11,12]. Contemporary judicial practice increasingly relies on standardized multidisciplinary medical evaluations performed by specialists in psychiatry, neurology, and forensic medicine to inform judicial decision-making while balancing individual protection with respect for self-determination [13,14,15,16]. Consequently, legal capacity assessment requires the integration of cognitive, functional, neurological, psychiatric, and medico-legal evidence rather than reliance on a single clinical finding or assessment tool. Although instruments such as the Mini-Mental State Examination (MMSE) and Activities of Daily Living (ADL) provide valuable functional information, they represent complementary components of a broader multidisciplinary assessment and cannot independently capture the complexity of legal capacity determination, particularly in individuals with dementia, intellectual disability, acquired brain injury, or psychiatric disorders [17,18,19,20,21,22,23].

In Türkiye, the Turkish Civil Code (TCC), largely derived from Swiss law, defines four principal legal grounds for guardianship under Articles 405, 406, 408, and 429. Within this legal framework, legal capacity assessments are routinely conducted by interdisciplinary medical boards comprising specialists in forensic medicine, psychiatry, and neurology. These boards provide standardized multidisciplinary medical evaluations that constitute the clinical basis for subsequent judicial decision-making. Despite its doctrinal alignment with Swiss legal principles, the Turkish system operates within a more centralized structure, where the implementation of voluntary or supported decision-making mechanisms remains limited [24,25,26].

Recent advances in large language models (LLMs) have created new opportunities for AI-assisted decision support in healthcare by enabling the synthesis of complex clinical, medico-legal, and narrative information within a unified analytical framework [27,28,29,30]. Rather than replacing expert judgment, LLMs have been increasingly investigated as tools to support structured reasoning, information integration, and consistency in complex decision-making tasks. These characteristics are particularly relevant to legal capacity assessment, where standardized multidisciplinary case information requires the integration of diverse clinical findings into a coherent assessment. Nevertheless, the application of LLMs in multidisciplinary medico-legal decision support remains insufficiently explored because of concerns regarding transparency, reliability, explainability, and human oversight [31,32]. While promising applications have been reported in forensic medicine, including postmortem interval estimation and toxicological classification, their potential role in standardized legal capacity assessment has received little investigation [33,34,35,36,37].

This study addresses this knowledge gap by evaluating the performance of state-of-the-art LLMs as AI-assisted decision-support tools using standardized medico-legal case vignettes, with interdisciplinary medical board (IMBD) assessments under the TCC serving as the reference standard. We hypothesized that standardized multidisciplinary case information and expert supervision would enable LLMs to demonstrate reproducible performance and high temporal stability when compared with the reference standard.

## 2. Methods

### 2.1. Study Design

The study used a retrospective method-comparison design to evaluate the performance of LLMs as AI-assisted decision-support tools for legal capacity assessment by comparing their outputs on standardized medico-legal case vignettes with IMBD assessments under the TCC, which served as the reference standard. The research team reviewed all court-ordered legal capacity assessments performed at a tertiary hospital between 1 January 2018, and 31 December 2024. The study was conducted in accordance with the TRIPOD-LLM reporting guidance [38]; the completed TRIPOD-LLM checklist is provided in Supplementary Table S1. This retrospective study was approved by the relevant institutional ethics committee (Decision No. 2025/148; 25 April 2025).

### 2.2. Participants and Criteria

Adult patients referred by Turkish civil courts for legal capacity assessment and evaluated by an interdisciplinary medical board consisting of one specialist each in forensic medicine, psychiatry, and neurology were eligible for inclusion. Eligibility required complete interdisciplinary medical board assessment records, documented neurological, psychiatric, and forensic diagnoses, and standardized cognitive and functional assessments (ADL and/or MMSE). Cases with incomplete records, repeat assessments, or missing key clinical information after quality checks were excluded. Of 241 eligible cases, 234 met the inclusion criteria and were included in the final analysis.

### 2.3. Data Sources and Structured Case Construction

Patient demographics, medical history, current medications, and medico-legal assessment findings were extracted from the interdisciplinary medical board assessment records. Cognitive and functional assessment results, including Activities of Daily Living (ADL; 0–100) and Mini-Mental State Examination (MMSE; 0–30) scores, were recorded when available. To ensure that all LLMs evaluated identical clinical information under identical assessment conditions, information from each real patient case was converted into a standardized, fully anonymized medico-legal case vignette before model evaluation. Two forensic medicine specialists independently reviewed each complete case file and extracted the relevant information using a predefined vignette template that included (1) patient demographics, (2) medical history and clinical diagnoses, (3) ADL and MMSE results, (4) current medications, and (5) essential medico-legal findings. The specialists independently prepared the structured case vignettes, after which discrepancies were resolved by consensus with a senior forensic medicine specialist. During extraction, the conclusion section of the IMBD report was masked so that the specialists preparing the vignettes were blinded to the final board recommendation at the time of data extraction. Diagnostic and functional findings were transcribed as descriptive clinical statements according to predefined extraction rules rather than copied verbatim, and conclusory or outcome-revealing wording from the original reports (for example, statements that the person was unable to manage his or her affairs, required continuous protection and care, or was suitable for guardianship) was excluded from the vignettes to prevent outcome leakage. Because all cases were referred by civil courts, the source records did not systematically document whether the person had personally requested guardianship; this element was therefore not represented in the vignettes. A blank copy of the vignette template is provided in Supplementary Table S2. All direct patient identifiers, court identifiers, and the IMBD assessment were removed before LLM evaluation to ensure that model outputs were generated solely from the standardized clinical and medico-legal information contained in each vignette. This standardized approach was designed to minimize presentation-related variability, facilitate fair comparison across LLMs, preserve patient confidentiality, and ensure that all models were evaluated using the same structured information under identical conditions.

### 2.4. Legal Framework

According to the TCC, the primary categorical outcome was the legal capacity category assigned in the signed IMBD assessment. The IMBD assessment represented the consensus medical opinion of one specialist each in forensic medicine, psychiatry, and neurology and was classified into one of five categories: Article 405 (guardianship due to mental disorder or deficiency), Article 406 (prodigality, addiction, or harmful lifestyle), Article 408 (old age, disability, or serious illness), Article 429 (appointment of a legal advisor; restricted legal capacity), or no guardianship. The signed IMBD assessment served as the reference standard for all model comparisons. The IMBD assessment constitutes an expert medical recommendation submitted to the referring court; the final legal determination of capacity is made exclusively by the judiciary. Accordingly, agreement with IMBD recommendations reflects concordance with an expert medical reference standard and should not be interpreted as agreement with final judicial decisions or legally correct outcomes.

### 2.5. Prompt Protocol

To ensure standardized and reproducible evaluation across all LLMs, a fixed prompt protocol was developed and applied without modification to every case and model. Each model was accessed through its publicly available web interface using the default settings in a newly initiated, isolated session without conversation history. The prompt consisted of four components presented in a fixed order: (1) an identical role instruction, (2) a standardized and fully anonymized medico-legal case vignette, (3) the full text of TCC Articles 405, 406, 408, and 429, and (4) a directive question. Consequently, every LLM received identical clinical information, identical legal provisions, identical prompt wording, and identical response instructions. This standardized protocol was designed to ensure that differences in model outputs reflected differences in model reasoning rather than variations in case presentation, legal information, or prompt formulation. Providing the complete text of the relevant TCC articles ensured that all models operated within an identical legal framework and minimized potential variability arising from differences in their pretrained legal knowledge. This approach was intended to reflect the standardized clinical and legal information routinely available to the interdisciplinary medical board during legal capacity assessments while ensuring that all models received identical inputs. All vignettes were fully anonymized before being entered into any third-party system, and no direct or indirect patient identifiers were transmitted. The institutional ethics approval covered the retrospective use of anonymized case records, including their evaluation with third-party AI systems, and chat history and model training options were disabled in the web interfaces where such settings were available, in accordance with institutional data-protection requirements.

An example of the standardized prompt template used throughout the study is shown below: *“You are an AI decision-support system assisting an interdisciplinary medical board composed of specialists in forensic medicine, psychiatry, and neurology. Your task is to review the following standardized medico-legal case and provide an AI-assisted medical recommendation regarding legal capacity according to the Turkish Civil Code (TCC).*

*Case: A 72-year-old woman was referred by the Civil Court of Peace for legal capacity assessment. She had a five-year history of Alzheimer’s disease and hypertension and was receiving donepezil (10 mg/day), memantine (20 mg/day), and quetiapine (25 mg/day). The standardized vignette included the findings of independent forensic medicine, neurology, and psychiatry examinations, together with the patient’s medical history, current medications, cognitive assessment (MMSE), functional assessment (ADL), and other relevant medico-legal information. Forensic medicine examination documented impaired orientation, difficulty comprehending the interview, and difficulty providing a consistent personal and medical history; neurological evaluation documented progressive cognitive decline; and psychiatric evaluation reported impaired insight and judgment. Her MMSE score was 17/30, and her ADL score was 45/100.*

*Article 405—Mental illness or mental deficiency: Every adult who, because of mental illness or mental deficiency, is unable to manage his or her own affairs, requires continuous assistance for protection and care, or endangers the safety of others shall be placed under guardianship.*

*Article 406—Prodigality, alcohol or drug addiction, harmful lifestyle, or mismanagement of property: Every adult who, because of prodigality, alcohol or drug addiction, a harmful lifestyle, or mismanagement of property, places himself or herself or the family at risk of poverty or deprivation, and therefore requires continuous protection and care or endangers the safety of others shall be placed under guardianship.*

*Article 408—Old age, disability, inexperience, or serious illness: Any adult who proves that he or she is unable to manage his or her own affairs because of old age, disability, inexperience, or serious illness may request to be placed under guardianship.*

*Article 429—Legal advisor: If there are insufficient grounds for guardianship but limitation of legal capacity is considered necessary for protection, a legal advisor may be appointed. The legal advisor shall be consulted for legal acts including litigation, settlement, transactions involving immovable property, negotiable instruments, extraordinary construction works, borrowing or lending, receipt of capital, donations, bills of exchange, and suretyship.*

*Question: Based on the standardized medico-legal case and the provisions of Articles 405, 406, 408, and 429 presented above, determine the most appropriate medical opinion regarding legal capacity. Select only one of the following categories: Article 405, Article 406, Article 408, Article 429, or No guardianship. Provide a concise justification for your selection.”*

All responses were generated under identical prompting conditions. The categorical recommendation constituted the primary model output, whereas the accompanying rationale was subsequently evaluated using the predefined explanation-quality rubric (Section 2.10) and incorporated into the human-factor, safety, and usability analyses. The complete prompt template is also reproduced in Supplementary Table S3. Because proceedings in cases ultimately classified under Article 408 may have been initiated upon an application by the person or a relative, whereas restriction under Article 408 legally depends on the person’s own request, and this information was not systematically available in the court-referred source records, the vignettes did not include a statement indicating whether the person had personally requested guardianship; the implications of this design feature are addressed in the Discussion and Limitations sections.

### 2.6. LLM Systems, Isolation and Retest Protocol

We evaluated ChatGPT-5.2 (OpenAI, San Francisco, CA, USA), Gemini 3 Pro (Google DeepMind, London, UK), and Claude 4.5 Sonnet (Anthropic, San Francisco, CA, USA). All initial model evaluations were conducted between 22 December 2025 and 4 January 2026, using zero-shot, fully isolated sessions without conversation history, external tools, or retrieval augmentation to minimize contamination and ensure consistency. To assess temporal stability, all 234 cases were re-evaluated under identical experimental conditions between 26 January 2026 and 8 February 2026. Model names, versions, prompts, and evaluation dates were systematically recorded to maximize reproducibility. All three systems were publicly available throughout both evaluation windows (GPT-5.2 released on 11 December 2025; Gemini 3 Pro on 18 November 2025; Claude Sonnet 4.5 on 29 September 2025), the model identifier displayed in each interface was verified at every session and remained unchanged across both windows, and no version transition was observed during the study period. Full details of the evaluated systems, including the model name displayed in each interface, access and subscription type, access dates, response settings, and session isolation, are summarized in Supplementary Table S4. Responses were not regenerated: the first complete response returned by each model was recorded, and in the rare event of a technical failure, refusal, or incomplete response, the case was resubmitted once in a new isolated session.

### 2.7. Output Coding and Blinded Adjudication

Raw model recommendations and accompanying rationales were extracted from each response. Recommendations were mapped to the five predefined outcome categories according to predefined coding rules (Supplementary Table S5). Two forensic medicine specialists, each with at least five years of experience, independently coded the model outputs and evaluated explanation quality using the pre-specified rubric (Section 2.10). Model identity (ChatGPT, Gemini, or Claude) was concealed from both raters throughout the coding process to minimize evaluation bias. Disagreements were resolved by consensus with a third forensic medicine specialist who also had at least five years of experience. Inter-rater agreement was assessed using Cohen’s κ for categorical classifications and ICC (2,k) for explanation-quality rubric scores.

### 2.8. Endpoints

The primary endpoint was concordance between AI-assisted LLM recommendations and IMBD recommendations for Article 405 versus all other outcome categories. Secondary endpoints included multi-class performance (accuracy, macro-F1, class-wise sensitivity, specificity, PPV, and NPV), calibration (Brier score, calibration intercept, and calibration slope), clinical utility assessed by decision-curve analysis (net benefit across threshold probabilities of 0.10–0.80), and test–retest reliability (percentage agreement and Cohen’s κ). Because the standardized prompt requested a single categorical recommendation with a concise justification, the LLMs did not directly generate probability estimates and were not asked to provide confidence scores. Predicted probabilities used in the discrimination, calibration, decision-curve, net reclassification, and integrated discrimination analyses were therefore obtained from logistic regression models fitted to the full analytic sample, in which the binary LLM recommendation for the relevant task (for example, Article 405 versus all other categories) served as the predictor, and the fitted probabilities from these models were treated as the predicted probabilities. Accordingly, all probability-based analyses in this study evaluate statistical models derived from the categorical LLM outputs rather than probabilities produced directly by the LLMs. Predictive modeling included logistic regression (Article 405 versus all other outcomes) and ordinal regression across ordered outcome categories, using dementia, ADL, age, and the LLM-derived recommendation as predictors. For ordinal modeling, outcome categories were ordered according to the degree of restriction of legal capacity imposed by each measure (no guardianship < Article 429 < Article 408 < Article 405); Article 406 was excluded from ordinal modeling because it occurred only once and reflects a legally distinct ground. This ordering reflects increasing intensity of legal intervention rather than a single clinical severity continuum, and the proportional-odds assumption was formally tested. Human-factor endpoints included System Usability Scale (SUS) scores, trust, explanation satisfaction (7-point Likert scale), and rework events under both evaluation conditions.

### 2.9. Usability and Trust Substudy

A clinician-focused substudy assessed professional acceptance using a stratified sample of 60 cases representing all outcome categories, selected by stratified random sampling across the five outcome categories. Two forensic medicine specialists, each with at least five years of experience, independently evaluated each case under two conditions: (1) standard vignette review and (2) AI-assisted review incorporating the LLM recommendation and accompanying rationale. After reviewing the AI-assisted output and before accessing the IMBD recommendation, both participants rated usability (System Usability Scale [SUS], 0–100), trust, and explanation satisfaction (7-point Likert scale). SUS scores of ≥68 were considered acceptable, whereas scores of ≥80 indicated excellent usability. The two review conditions were completed in separate sessions, with the standard vignette review performed first and the AI-assisted review performed after a washout interval during which case order was re-randomized to reduce recall effects; because condition order was not counterbalanced, residual order and learning effects cannot be excluded. A rework event was defined as any revision of an initially recorded recommendation by the reviewing specialist before finalization of the case assessment. The substudy assessed the impact of AI-assisted decision support on clinician confidence, perceived usability, trust, explanation satisfaction, and decision-making while preserving independent expert judgment. Given the small number of participating specialists, the human-factor findings should be regarded as exploratory.

### 2.10. Explanation Quality Rubric and Safety Checks

Each LLM generated not only a categorical recommendation but also an accompanying rationale explicitly linking the clinical findings to the relevant TCC criteria, as specified in the standardized prompt. Two forensic medicine specialists, each with at least five years of experience and blinded to model identity, independently evaluated each rationale using a pre-specified 0–6-point rubric (Supplementary Table S5; 0–2 points each for alignment with the applicable TCC article, consideration of the least restrictive alternative, and acknowledgment of uncertainty or missing clinical information). Disagreements were resolved by a third forensic medicine specialist with at least five years of experience. Predefined safety flags, established before analysis, were triggered when Article 405 was recommended without clear cognitive or functional justification or when less restrictive alternatives (e.g., Article 429 or no guardianship) were not adequately considered. Safety flags were counted at the level of individual model outputs and were additionally summarized at the case level, with a case considered flagged if at least one of its model outputs met a predefined flag criterion. Flagged cases underwent mandatory review by a senior forensic medicine specialist to ensure medico-legal proportionality and appropriate human oversight.

### 2.11. Handling Missing Data and Class Imbalance

Missing MMSE values were handled using multiple imputation by chained equations (MICE; *m* = 20, 30 iterations), incorporating age, sex, diagnoses, ADL, medications, and the IMBD recommendation. Convergence was assessed using trace plots, and parameter estimates were pooled according to Rubin’s rules. Because Article 406 was infrequent, descriptive reporting with macro-averaged performance metrics, class-balanced accuracy, and case-weighted bootstrapping was applied. A sensitivity analysis using complete-case logistic regression (*n* = 68) was performed alongside the pooled MICE estimates.

### 2.12. Calibration and Internal Validation

For each model and each relevant binary classification task (e.g., Article 405 versus all other outcomes), we calculated the Brier score, calibration intercept, and calibration slope. When calibration was suboptimal, post hoc calibration using isotonic regression was performed. Performance uncertainty and pairwise model comparisons were estimated using bias-corrected bootstrap resampling (1000 iterations).

### 2.13. Decision Thresholds, Net Benefit, and Reclassification

Before analysis, predicted probabilities were mapped to operational decision thresholds reflecting routine IMBD practice: ≥0.70, strongly consider Article 405; 0.25–0.69, enhanced expert review with consideration of less restrictive alternatives; and <0.25, generally no guardianship. Net benefit across threshold probabilities was assessed using decision-curve analysis. We evaluated the incremental clinical value of adding the LLM recommendation to the clinical baseline model (dementia, ADL, and age) using ΔAUC, Akaike Information Criterion (AIC), Net Reclassification Improvement (NRI), and Integrated Discrimination Improvement (IDI).

### 2.14. Statistical Analysis

Continuous variables were summarized as mean ± SD or median [IQR], and categorical variables as counts (%). Group comparisons were performed using appropriate parametric and non-parametric tests (Student’s *t*-test, Mann–Whitney *U* test, χ^2^ test, or Fisher’s exact test), as appropriate. Discrimination was assessed using the area under the receiver operating characteristic curve (AUC) with DeLong 95% confidence intervals, and multi-class performance was evaluated using macro-F1 and class-wise performance metrics. Agreement was assessed using Cohen’s κ and ICC (2,k), and correlations were evaluated using Spearman’s ρ. Paired model comparisons were performed using McNemar’s test and the Friedman test with Holm correction for multiple comparisons. The usability substudy used non-parametric within-clinician analyses (Wilcoxon signed-rank test and Friedman test). All statistical analyses were performed using R (version 4.3).

### 2.15. Human in the Loop Governance and Deployment Principles

LLM outputs were treated exclusively as AI-assisted decision-support recommendations and did not replace expert clinical or legal judgment. Final medico-legal opinions remained the responsibility of the interdisciplinary medical board. Mandatory senior review was required for Article 405 recommendations without clear cognitive or functional justification or when less restrictive alternatives were not adequately considered. Cases with discordant or low-confidence outputs required dual-expert adjudication before final reporting to ensure appropriate human oversight and medico-legal proportionality.

## 3. Results

### 3.1. Sample Characteristics and Clinical Profile

A total of 234 adults referred by Turkish civil courts were included (mean age 64.3 ± 25.1 years; median 74.2, IQR 43.5–84.9); 53.4% (*n* = 125) were women. Dementia was the most prevalent diagnosis (47.9%, 112/234), followed by intellectual disability (19.2%, 45/234) and organic brain injury (6.0%, 14/234). Other individual conditions, including schizophrenia, bipolar disorder, stroke sequelae, Parkinson’s disease, and epilepsy, each occurred in <4% of referrals.

Neurological examination confirmed dementia in 48.3% (113/234) and yielded no abnormality in 26.5% (62/234). Stroke sequelae (5.1%), epilepsy (3.8%), mild intellectual disability (4.3%), organic brain injury (4.3%), and Parkinson’s disease (1.3%) were infrequent. Psychiatric assessment identified no formal diagnosis in 69.7% (163/234). When present, intellectual disability predominated (mild 5.1%, moderate 4.7%, severe 4.3%). Other psychiatric disorders were uncommon: schizophrenia (3.0%), bipolar disorder (1.3%), anxiety disorders (3.4%), major depression (2.6%), and substance-use disorder (0.4%). Small discrepancies in totals reflect occasional dual documentation and rounding.

Prior medical records indicated no documented neurological/psychiatric condition in 27.4% (64/234). Among documented comorbidities, dementia was most common (20.1%, 47/234), followed by stroke (15.0%, 35/234) and Parkinson’s disease (6.4%, 15/234). Intellectual disability accounted for 13.1%, whereas primary psychotic disorders comprised 2.6%.

Most individuals (81.6%, 191/234) were medication-free or had no documented pharmacotherapy at assessment. Among 43 treated patients, antipsychotics were most frequent (4.3%, 10/234), followed by dementia drugs (3.8%, 9/234), antiepileptics (3.4%, 8/234), and antidepressants (2.6%, 6/234). Polytherapy was rare (3.4%, 8/234).

MMSE scores were available for 28.6% (67/234), with a mean of 15.0 ± 7.2 (median 15). ADL scores covered the full range (0–100; mean 51.4 ± 35.6, median 45). Severe dependence (ADL ≤ 25) was present in 34.2% (80/234), while full independence (ADL ≥ 76) applied to 33.3% (78/234). MMSE moderately correlated with ADL (Spearman r = 0.47, *p* < 0.001). Age negatively correlated with ADL (r = −0.37, *p* < 0.001) but not with MMSE (r = −0.12, *p* = 0.33).

### 3.2. Multidisciplinary Board Decisions and Clinical Predictors

Board recommendations were distributed across five outcome categories:

- TCC Article 405 (full guardianship): 154/234 (65.8%);
- TCC Article 408 (age/disability/serious illness): 27/234 (11.5%);
- TCC Article 429 (restricted capacity/legal advisor): 18/234 (7.7%);
- No guardianship: 34/234 (14.5%);
- TCC Article 406 (prodigality/addiction): 1/234 (0.4%).

Diagnostic category strongly predicted IMBD outcome with a large and highly significant association in both specialties (forensic: χ^2^(64) = 576.8, Cramér’s V = 0.63; psychiatric: χ^2^(56) = 396.9, V = 0.55; both *p* < 10^−56^). Dementia cases were predominantly classified under Article 405 (91.1%, 102/112). Individuals without a documented diagnosis were most often classified as requiring no guardianship (61.5%, 24/39) or placed under Article 408 (35.9%, 14/39). Mild intellectual disability typically resulted in Article 429 (75.0%, 9/12), whereas moderate-to-severe forms nearly always led to Article 405 (95.8%, 23/24). Sex showed no effect; age did not influence the outcome (χ^2^(6) = 1.88, *p* = 0.76).

A binary logistic regression contrasting Article 405 vs. all others (*n* = 68 complete cases) showed that dementia increased the odds of an Article 405 recommendation 6.5-fold (OR = 6.5, 95% CI 1.2–35.2, *p* = 0.029), while each ADL point decreased the odds by 4% (OR = 0.96, 95% CI 0.94–0.99, *p* = 0.004). Model discrimination was excellent (AUC = 0.87, 95% CI 0.80–0.93).

Ordinal regression of the four ordered outcome categories specified in Section 2.8 (no guardianship < Article 429 < Article 408 < Article 405, with the single Article 406 case excluded; after multiple imputation of missing MMSE) confirmed these patterns: dementia markedly increased the likelihood of stricter rulings (≈29-fold, *p* < 0.001), and higher ADL remained protective (~4% per point, *p* < 0.001). MICE convergence diagnostics indicated stable estimates across imputations. Proportional-odds assumptions were met, and sensitivity analyses were consistent. Because Article 406 occurred only once (1/234), results for this class are reported descriptively only, and per-class inferential estimates were not computed. A sensitivity analysis using complete-case data yielded consistent effect sizes.

### 3.3. Performance, Stability, and Calibration of Zero-Shot LLMs

Overall agreement between LLM predictions and IMBD outcomes was substantial (Cohen’s κ = 0.60–0.73). Gemini 3 Pro achieved the highest overall accuracy (86.3%), while ChatGPT-5.2 demonstrated the best class balance (macro-F1 = 0.79) and the highest AUC for Article 405 versus the rest (AUC = 0.91, 95% CI 0.86–0.95) (Table 1). Confusion matrices for each model against the IMBD reference standard are provided in Supplementary Table S6. Across models, sensitivity for Article 405 ranged from 0.88 to 0.94 and specificity from 0.82 to 0.90; PPV ranged from 0.92 to 0.95 and NPV from 0.78 to 0.85.

Class-specific sensitivity (recall) was high for Article 405 (≥93%) and no guardianship (≥91%) but lowest for Article 408 (~30%), which was frequently misclassified as Article 405. Gemini 3 Pro outperformed the other models for Article 429 (77.8%), whereas ChatGPT-5.2 and Claude 4.5 Sonnet misclassified most cases (Table 2). Because Article 406 occurred in only a single case, the corresponding values are descriptive only and should not be interpreted as stable performance.

Test–retest analysis of all 234 cases after 30 days showed near-perfect temporal stability: Gemini 3 Pro repeated 97% of outputs (κ = 0.95), while ChatGPT-5.2 and Claude 4.5 Sonnet each reached 94% consistency (κ = 0.87) (Table 3). These findings indicate minimal stochastic drift over the 30-day retest interval.

Pairwise McNemar tests and a three-way Friedman test (Supplementary Table S7) demonstrated no significant difference in misclassification rates across models after Holm correction (all adjusted *p* ≥ 0.30). Consistent with the pre-specified 0.10–0.80 evaluation range, positive net benefit was observed primarily within clinically relevant 0.25–0.70 thresholds.

Calibration and decision-curve analyses, conducted as secondary statistical analyses of the logistic models derived from the categorical LLM outputs (Section 2.8), indicated acceptable probabilistic performance. Brier scores were lowest for ChatGPT-5.2 in Articles 405 and 408 (0.086 and 0.156) and for Gemini 3 Pro in Article 429 (0.128). Decision-curve analysis showed positive net clinical benefit for ChatGPT-5.2 across thresholds 0.25–0.70 for Article 405, and for Gemini 3 Pro at higher thresholds, including the only positive net benefit for Article 429 (0.30–0.65). Calibration intercepts were near 0 (–0.11 to 0.00) and slopes were close to 1 for ChatGPT-5.2 and Gemini 3 Pro (0.98–1.12), with modest overconfidence for Claude 4.5 Sonnet (1.20–1.27). These estimates characterize the derived statistical models rather than probabilities produced by the LLMs themselves, and the associated calibration and net-benefit findings should be interpreted cautiously pending external validation. All calibration metrics reported in Supplementary Table S8 are final estimates obtained after post hoc isotonic recalibration of the logistic models described in Section 2.8; pre-recalibration values are not reported, and recalibration was performed on the full analytic sample.

Adding a binary indicator (“ChatGPT-5.2 predicts TCC Article 405”) to the clinical baseline (dementia + ADL + age) reduced AIC by 12, increased AUC from 0.87 (95% CI 0.80–0.93) to 0.93 (95% CI 0.89–0.97), and improved reclassification (NRI = 0.21, IDI = 0.07). The indicator increased the odds of an IMBD recommendation under Article 405 by 5.6-fold (95% CI 2.1–15.0, *p* < 0.001). Dementia (OR ≈ 6.4) and lower ADL (OR ≈ 0.96 per point) remained significant predictors.

### 3.4. Subgroup Analyses

Model performance was consistent across sex and age strata. Accuracy and Cohen’s κ values did not differ significantly between individuals aged ≤65 and >65 or between men and women (all *p* > 0.10). Among cases with available MMSE, performance was slightly higher when MMSE was >18 than when it was ≤18, yet differences were small and not statistically significant. No statistically detectable differences were observed in the examined age and sex subgroups, although the analysis was not powered to exclude smaller subgroup effects.

### 3.5. Human Factors Evaluation

Among the 60-case stratified subsample, the participating forensic medicine specialists rated LLM-assisted reviews as moderately usable and trustworthy. The mean SUS score was 72.4 ± 8.6, indicating “good” usability. Perceived trust averaged 5.0/7, and explanation satisfaction 5.1/7. The mean explanation-quality score was 4.8/6 (IQR 4–6), with 84.2% of outputs meeting the predefined adequacy threshold (≥4). Inter-rater reliability for rationale scoring was high (ICC (2,k) = 0.79–0.86). Rework events occurred in 6.7% of assisted reviews versus 13.3% of standard reviews. Within-clinician comparisons showed significant improvement with LLM assistance versus standard review (paired Wilcoxon *p* < 0.01), and no significant difference among models in the assisted condition (Friedman *p* = 0.33). Safety checks flagged 4 individual model outputs, corresponding to 4 of 234 cases (1.7% of cases; 4 of 702 model outputs, 0.6%), for lacking functional/cognitive justification or ignoring less-restrictive alternatives (representative anonymized model outputs, including a flagged output, are provided in Supplementary Table S9); all flagged cases underwent mandatory senior review before finalization. In addition, dual-expert adjudication was invoked whenever model recommendations were discordant or confidence was explicitly low, in line with the pre-specified governance plan.

## 4. Discussion

### 4.1. Cross Jurisdictional Relevance

TCC-based outcomes correspond to established guardianship frameworks across multiple jurisdictions. Article 405 reflects full guardianship, Article 429 reflects supported or limited decision-making [39], Article 408 reflects protective measures related to aging or serious illness, and no guardianship reflects the preservation of legal capacity. This alignment is approximate and conceptual rather than legally exact, because guardianship, supported decision-making, legal capacity restrictions, and advisor systems are not fully equivalent across the Turkish, UK, German, and Swiss legal systems (Supplementary Table S10); with this caveat, the TCC-based classification framework can be conceptually related to comparable legal systems, including the UK Mental Capacity Act and the German Betreuungsrecht [40,41]. These findings are consistent with Article 12 of the United Nations Convention on the Rights of Persons with Disabilities (CRPD), particularly the principles of equal legal recognition and least-restrictive intervention. The observed agreement between LLM recommendations and IMBD recommendations suggests that structured AI-assisted clinical decision support may be adaptable to comparable legal capacity assessment frameworks, provided that jurisdiction-specific legal standards and expert oversight are maintained. Accordingly, the TCC-based framework may serve as a useful conceptual model for evaluating AI-assisted decision support in jurisdictions with comparable guardianship principles, including the United Kingdom, Germany, and Switzerland, while requiring adaptation to local legal requirements [40,41,42]. Because the present data derive from a single Turkish institution and a single legal system, the conclusions of this study pertain primarily to the Turkish Civil Code setting, and any extension to other jurisdictions should be regarded as hypothesis-generating.

### 4.2. Clinical Impact and Utility

The evaluated LLMs demonstrated high agreement with IMBD recommendations, achieving overall accuracies of up to 86.3%, consistent with previous evidence that LLMs can support structured clinical reasoning and decision support [43,44,45]. These findings support the potential role of LLMs as AI-assisted clinical decision support systems for legal capacity assessment, particularly in multidisciplinary medico-legal practice. In addition, LLM-assisted review was associated with fewer decision revisions in the usability substudy (13.3% versus 6.7%); given the small number of evaluators and the exploratory design of the substudy, this observation should be interpreted cautiously as a preliminary signal of improved decision consistency rather than established workflow efficiency [46,47,48,49].

Decision-curve analysis demonstrated positive net clinical benefit across clinically relevant threshold probabilities (0.25–0.70), suggesting potential clinical utility for identifying cases requiring full guardianship while minimizing unnecessary restrictions. The improvement in discrimination after incorporating LLM recommendations into the clinical baseline model (ΔAUC = +0.06) further indicates that LLM-derived recommendations provide incremental value beyond conventional clinical predictors. Collectively, these findings support the potential use of LLMs for initial case prioritization and structured decision support while preserving final clinical responsibility within the interdisciplinary medical board [50,51].

Performance was lower for Article 408, highlighting the inherent complexity of evaluating partial functional impairment while preserving individual autonomy. This finding indicates that proportionality-based decisions require more granular functional information than diagnostic labels alone can provide. Future development may benefit from incorporating richer functional assessments, domain-specific training, and explicit evaluation of residual decision-making capacity. The absence of downstream outcome evaluation and the persistent uncertainty observed in borderline autonomy cases further support the continued need for expert human oversight.

### 4.3. LLM Vulnerabilities in Borderline Autonomy Cases

Although structured prompting and explanation-constrained outputs improved overall reliability, performance remained lower in borderline legal capacity assessments, particularly for cases evaluated under Article 408. These cases require nuanced assessment of residual decision-making capacity, where functional impairment coexists with partially preserved autonomy and the appropriate legal recommendation depends on proportionality rather than diagnosis alone.

The reduced performance observed for Article 408 suggests that current LLMs tend to place greater emphasis on diagnostic labels and functional impairment than on preserved decision-making capacity and the principle of the least restrictive intervention. This interpretation is consistent with the predominant misclassification pattern observed in the present study, in which Article 408 cases were most frequently classified as Article 405. This misclassification pattern should be regarded not merely as a technical limitation but as a clinically, ethically, and medico-legally significant failure mode: recommending Article 405 for a person whose situation corresponds to Article 408 replaces a request-based, proportionate protective measure with full guardianship, thereby unnecessarily restricting autonomy in precisely the cases in which the law is designed to preserve it. An additional contributing factor may be that the vignettes, reflecting the court-referred source records, did not contain information on whether the person had personally requested guardianship, which is a constitutive element of Article 408; the models may therefore have lacked a legally decisive criterion for distinguishing Article 408 from Article 405. Accordingly, the weak Article 408 performance most plausibly reflects the combination of intrinsic model limitations in weighing proportionality and partially preserved autonomy with the absence of the person’s own request from the vignettes; neither factor alone fully accounts for the observed error pattern. In any operational deployment, outputs recommending Article 405 in clinically borderline cases would therefore require mandatory expert review.

These findings indicate that proportionality-based medico-legal judgments remain particularly challenging for current LLMs. Such assessments require integration of cognitive status, functional ability, clinical context, and legal principles rather than reliance on diagnostic information alone. Future development may therefore benefit from incorporating richer functional assessments, explicit evaluation of residual decision-making capacity, and jurisdiction-specific legal reasoning into AI-assisted clinical decision support systems.

Accordingly, AI-assisted clinical decision support for legal capacity assessment should incorporate explicit proportionality safeguards, transparent communication of uncertainty, and mandatory expert review for cases involving borderline autonomy or partial functional impairment. These findings reinforce that LLMs should support, rather than replace, interdisciplinary medico-legal decision-making.

### 4.4. Methodological Strengths and Safeguards

The present study incorporated multiple methodological safeguards to enhance the reliability, transparency, and reproducibility of AI-assisted clinical decision support for legal capacity assessment [43]. Test–retest analysis demonstrated high temporal stability across all evaluated models (94–97% agreement over a one-month interval), supporting the reproducibility of LLM-generated recommendations.

A further consideration specific to the legal domain is the sensitivity of legal language to wording and syntax. In statutory interpretation, changing a single word, a modal expression, or the order of clauses within a sentence can substantially alter the meaning of a provision and, consequently, the decision that follows from it; the distinction between guardianship due to mental illness under Article 405 and guardianship upon the person’s own request under Article 408 ultimately rests on precisely such fine-grained textual elements. Because LLMs generate outputs through probabilistic language modeling, they may respond to such micro-level linguistic variations in ways that do not necessarily follow legal interpretive canons, and sensitivity of model outputs to minor changes in prompt wording is a recognized property of these systems. In the present study, this risk was mitigated, although not eliminated, by presenting the statutory texts verbatim and holding the standardized prompt template fixed across all cases and models; the robustness of model recommendations to paraphrases of the vignettes or of the legal provisions was not evaluated and should not be assumed.

Calibration, decision-curve analysis, and blinded explanation assessment complemented conventional discrimination metrics, providing a broader evaluation of clinical utility, reliability, and explainability. Most model-generated rationales adequately linked clinical findings to the relevant legal framework while considering less restrictive alternatives, with 84% of explanations achieving satisfactory quality (≥4/6) [52].

Methodological safeguards extended beyond predictive performance to include structured safety monitoring. Potentially over-restrictive recommendations and failures to consider less restrictive alternatives were identified in only 1.7% of cases, and all were reviewed through mandatory senior expert oversight, supporting transparent and proportionate medico-legal decision-making.

Missing data and class imbalance were addressed using multiple imputation and complementary performance metrics, thereby improving the robustness of model evaluation for infrequent outcome categories. Adherence to the TRIPOD-LLM reporting guideline further strengthened methodological transparency and reproducibility.

Collectively, these findings demonstrate that rigorous methodological design is as important as predictive performance when evaluating LLMs for high-risk medico-legal applications. Accordingly, implementation of AI-assisted clinical decision support in legal capacity assessment should be based on rigorous methodological validation, transparent reasoning, calibration, explainability, and continuous expert oversight rather than predictive performance alone [44,53,54].

## 5. Limitations

This study has several limitations that should be considered when interpreting the findings. First, the retrospective design evaluated AI-assisted LLM performance under standardized simulated conditions and therefore could not determine the real-world impact of AI-assisted clinical decision support on downstream outcomes such as judicial decisions, implementation of guardianship measures, or patient-centered outcomes.

Second, although the reference standard was based on interdisciplinary medical board recommendations, the final legal determination is made by the courts. Consequently, the influence of AI-assisted medical recommendations on judicial decision-making was not evaluated and was therefore beyond the scope of the present study.

Third, the study was conducted within a single national legal and healthcare system, which may limit the generalizability of the findings to jurisdictions with different legislative frameworks, clinical practices, or institutional structures.

Fourth, the models were accessed through publicly available web interfaces, and commercial LLMs are subject to undisclosed updates over time; although evaluation dates were recorded and test–retest stability was high over a one-month interval, model version drift cannot be excluded and may affect the reproducibility of the reported results.

Fifth, because the models produced only categorical recommendations, all probability-based analyses (AUC, calibration, decision-curve analysis, NRI, and IDI) were derived from secondary logistic models fitted to the study data rather than from probabilities generated directly by the LLMs, and these estimates were internally recalibrated without external validation in an independent cohort.

Sixth, although conclusory wording was excluded from the vignettes according to predefined extraction rules, subtle residual outcome leakage through the phrasing of clinical findings cannot be completely ruled out and could inflate apparent model performance.

Seventh, MMSE scores were available for only 28.6% of cases and required multiple imputation, and the outcome distribution was markedly imbalanced: Article 406 occurred only once, precluding any performance inference for this category, and performance for Article 408 was weak, likely reflecting both intrinsic model limitations and the absence of the person’s request information from the vignettes, which limits conclusions for exactly the borderline categories in which decision support would be most valuable.

Eighth, the human-factor substudy involved only two forensic medicine specialists and a 60-case subsample without counterbalanced condition order, so the usability, trust, and rework findings are exploratory and cannot be generalized to other evaluators or settings.

Finally, LLMs were evaluated under standardized zero-shot conditions using retrospective case vignettes, which cannot fully reproduce real-world assessment conditions, and without iterative user interaction or retrieval augmentation. In addition, whether the models would produce the same recommendations if the case descriptions or statutory passages were slightly reworded was not examined, although in legal language even small lexical or syntactic changes can alter meaning and legal consequences. Future studies should investigate model performance within prospective clinical workflows, jurisdiction-specific legal frameworks, and real-world human–AI collaboration to better understand the effectiveness of interactive AI-assisted decision-support systems.

## 6. Conclusions

This study demonstrates that, when evaluated under standardized prompting conditions using identical medico-legal case vignettes and a shared legal framework, contemporary large language models can provide reproducible and clinically coherent AI-assisted recommendations that show substantial agreement with interdisciplinary medical board assessments. Rather than functioning as autonomous decision-makers, the evaluated LLMs demonstrated their greatest potential as structured decision-support systems that assist in integrating multidisciplinary clinical and medico-legal information within a standardized assessment process.

At the same time, reduced performance in Article 408 and other borderline autonomy cases indicates that proportionality-based legal capacity assessments remain challenging for current LLMs. These findings reinforce that legal capacity assessment requires interdisciplinary clinical judgment and careful application of jurisdiction-specific legal principles, particularly when less restrictive alternatives must be considered.

Accordingly, the findings of this study support the further investigation of LLMs as AI-assisted decision-support tools for legal capacity assessment, provided that they operate within standardized evaluation protocols, transparent reasoning processes, predefined safety safeguards, and continuous expert human oversight. The present findings do not establish readiness for routine medico-legal implementation: the weak performance in Article 408 cases, the retrospective vignette-based design, the derivation of probability-based metrics from secondary statistical models, and the absence of external validation all require resolution through prospective evaluation before any clinical or medico-legal deployment. Under these conditions, LLMs may contribute to improving consistency, transparency, and standardization in multidisciplinary medico-legal assessments without replacing clinical or judicial decision-making.

We nevertheless emphasize the risks inherent in applying artificial intelligence to the legal system and to sensitive fields such as medicine. In the legal domain, even the change of a single word or of the order of a sentence can alter meaning and the resulting decision, and erroneous, overconfident, or unverifiable model outputs may translate into unjustified restrictions of legal capacity and autonomy. Fluent but incorrect justifications may additionally induce automation bias in expert users, and systematic algorithmic errors may propagate across large numbers of cases. In fields where decisions directly affect fundamental rights, AI systems should therefore be used, if at all, exclusively as auxiliary decision-support tools within legally accountable, expert-controlled frameworks, and never as autonomous decision-makers.

Future prospective multicenter studies across different legal systems are warranted to determine the effectiveness, safety, and generalizability of AI-assisted decision support within routine legal capacity assessment.

## Figures and Tables

**Table 1:** Overall metrics comparing zero-shot LLM predictions to IMBD recommendations.

*Metric*	*ChatGPT-5.2*	*Gemini 3 Pro*	*Claude 4.5 Sonnet*
*Accuracy % (95% CI)*	82.5 (77.1–87.0)	86.3 (81.3–90.3)	79.5 (73.7–84.4)
*Macro-F1*	0.79	0.75	0.71
*Cohen’s κ*	0.64	0.73	0.60
*AUC, “TCC Article 405 vs. rest”*	0.91	0.88	0.86

**Table 2:** Class-specific sensitivity (recall) of language model predictions versus IMBD recommendations. Values for Article 406 are based on a single case and are descriptive only.

*TCC Article (n)*	*ChatGPT-5.2*	*Gemini 3 Pro*	*Claude 4.5 Sonnet*
*405 (154)*	97.4%	95.5%	92.9%
*406 (1)*	100%	100%	100%
*408 (27)*	29.6%	29.6%	22.2%
*429 (18)*	16.7%	77.8%	16.7%
*No guardianship (34)*	91.2%	94.1%	97.1%

Error pattern: Misclassifications clustered at the TCC Article 405 ↔ TCC Article 408 threshold (≈66% of all errors) and, to a lesser extent, at TCC Article 429. Age and sex were not associated with error frequency (χ^2^, *p* > 0.40).

**Table 3:** One-month test–retest consistency of LLM outputs.

*LLMs*	*Exact Match %*	*Cohen’s κ*
*ChatGPT-5.2*	94	0.87
*Gemini 3 Pro*	97	0.95
*Claude 4.5 Sonnet*	94	0.87

## Data Availability

The datasets generated and/or analyzed during the current study are not publicly available because they contain anonymized clinical and medico-legal data subject to ethical, legal, and institutional restrictions.

## Supplementary Materials

The following supporting information can be downloaded at: https://www.mdpi.com/article/10.3390/healthcare14152261/s1, Supplementary Table S1: TRIPOD-LLM checklist. Supplementary Table S2: Blank standardized vignette template used for structured case construction. No patient-identifying information was recorded at any stage; the IMBD conclusion was excluded from all vignettes. Supplementary Table S3: Complete standardized prompt template applied identically to all models and cases. Supplementary Table S4: Access details and configuration of the evaluated LLM systems. Supplementary Table S5: Output coding rules (Part A) and explanation-quality rubric (Part B). Supplementary Table S6: Confusion matrices for each LLM versus IMBD recommendations at first-round zero-shot evaluation (rows: IMBD reference category; columns: model prediction; *n* = 234). Supplementary Table S7: Statistical comparison of classification errors across LLMs (McNemar and Friedman tests). Supplementary Table S8: Final calibration metrics following post hoc isotonic recalibration and decision-curve analysis for the binary classification tasks (TCC Articles 405, 408, and 429). Supplementary Table S9: Representative anonymized model outputs. Case details are condensed and fully anonymized; outputs are shown as recorded, lightly edited only for anonymization and length. Supplementary Table S10: Cross-jurisdictional comparison of guardianship frameworks under the Turkish Civil Code (TCC), the UK Mental Capacity Act 2005, the German Civil Code (BGB), and the Swiss Civil Code (ZGB).

## Abbreviations

The following abbreviations are used in this manuscript:

ADL	Activities of Daily Living
AIC	Akaike Information Criterion
AI	Artificial Intelligence
AUC	Area Under the Receiver Operating Characteristic Curve
CI	Confidence Interval
CRPD	Convention on the Rights of Persons with Disabilities
DCA	Decision-Curve Analysis
df	Degrees of Freedom
HITL	Human-in-the-Loop
ICC	Intraclass Correlation Coefficient
IDI	Integrated Discrimination Improvement
IMBD	Interdisciplinary Medical Board
IQR	Interquartile Range
LLM	Large Language Model
MCA	Mental Capacity Act 2005 (United Kingdom)
MICE	Multiple Imputation by Chained Equations
MMSE	Mini-Mental State Examination
NPV	Negative Predictive Value
NRI	Net Reclassification Improvement
OR	Odds Ratio
PPV	Positive Predictive Value
ROC	Receiver Operating Characteristic
SD	Standard Deviation
SUS	System Usability Scale
TCC	Turkish Civil Code
TRIPOD-LLM	Transparent Reporting of a Multivariable Prediction Model for Individual Prognosis or Diagnosis–LLM Extension
UK	United Kingdom
