# Supplementary material for: Evaluating Large Language Models for AI-Assisted Decision Support in Legal Capacity Assessment: A Comparative Study Using Interdisciplinary Medical Board Recommendations as the Expert Medical Reference Standard
Source: Healthcare (Basel). 2026 Jul 24;14(15):2261. doi: 10.3390/healthcare14152261 (PMC13465600; doi:10.3390/healthcare14152261)
Supplement: Supplementary file 1 [file healthcare-14-02261-s001.zip › healthcare-4443668-supplementary.pdf]

Supplementary Materials

Evaluating Large Language Models for AI-Assisted Decision Support in Legal Capacity Assessment: A Comparative Study Using Interdisciplinary Medical Board Recommendations as the Expert Medical Reference Standard

Supplementary Table S1: TRIPOD-LLM checklist

TRIPOD-LLM domain / item	Description	Reported in
Title	Identify the study as developing/evaluating an LLM; specify task and setting	Title
Abstract	Structured summary of objectives, data, LLMs, evaluation, and results	Abstract
Background	Healthcare context, rationale, and role of the LLM	Section 1
Objectives	Study objectives and hypotheses	Section 1
Data sources	Description of source data, setting, and dates	Sections 2.1–2.3
Participants	Eligibility criteria and participant flow	Section 2.2
Outcome	Definition of the reference standard and outcome categories	Section 2.4
LLM identification	Name, version, developer, and access route of each LLM	Section 2.6; Table S4
Prompting strategy	Full prompt(s), prompt development, and standardization	Section 2.5; Table S3
Inference settings	Interface settings, session isolation, and regeneration policy	Section 2.6; Table S4
Fine-tuning / adaptation	Statement that no fine-tuning or retrieval augmentation was used	Section 2.6
Output processing	Rules for mapping raw outputs to analyzable categories	Section 2.7; Table S5
Human evaluation	Blinded dual coding, adjudication, and explanation-quality rubric	Sections 2.7, 2.10; Table S5
Sample size	How the analytic sample was determined	Section 2.2
Missing data	Handling of missing data (MICE) and sensitivity analysis	Section 2.11
Statistical methods	Performance metrics, probability derivation, calibration, DCA, and comparisons	Sections 2.8, 2.12–2.14
Reliability	Test–retest protocol and inter-rater agreement	Sections 2.6, 2.7; Table 3
Participants (results)	Sample characteristics and outcome distribution	Sections 3.1–3.2
Model performance	Discrimination, class-specific performance, and confusion matrices	Section 3.3; Tables 1–2; Table S6
Calibration and utility	Calibration metrics and decision-curve analysis	Section 3.3; Table S8
Subgroup analyses	Performance across age and sex strata	Section 3.4
Human factors and safety	Usability, trust, rework events, and safety flags	Section 3.5; Table S9
Interpretation	Interpretation in context of evidence and legal setting	Sections 4.1–4.4; Table S10
Limitations	Study limitations, including LLM-specific limitations	Section 5
Implications	Implications for practice and future research	Sections 4.3, 6

Ethics and governance	Ethics approval, data protection, and human oversight	Sections 2.1, 2.5, 2.15
Funding and conflicts	Funding source and conflicts of interest	Back matter
Data availability	Availability of data and materials	Back matter

Supplementary Table S2: Blank standardized vignette template used for structured case construction. No patient-identifying information was recorded at any stage; the IMBD conclusion was excluded from all vignettes.

**Vignette field**	**Content entered (blank template)**
Study case code	[Non-identifying sequential study code]
1. Demographics	Age (years): [ ] Sex: [ ]
Referral context	Referred by the Civil Court of Peace for legal capacity assessment
2. Medical history and clinical diagnoses	[Documented diagnoses and relevant medical history, transcribed as descriptive clinical statements]
3a. Cognitive assessment	MMSE (0–30): [score or "not available"]
3b. Functional assessment	ADL (0–100): [score]
4. Current medications	[Drug names and daily doses, or "no documented pharmacotherapy"]
5a. Forensic medicine examination findings	[Descriptive findings; conclusory or outcome-revealing wording excluded]
5b. Neurological examination findings	[Descriptive findings; conclusory or outcome-revealing wording excluded]
5c. Psychiatric examination findings	[Descriptive findings; conclusory or outcome-revealing wording excluded]
5d. Other essential medico-legal findings	[Descriptive findings only]
Excluded by design	All direct patient identifiers; court file identifiers; the IMBD assessment and conclusion; any statement of the person’s request for guardianship (not systematically documented in court-referred records)

Note: Bracketed entries indicate the fields completed for each case; this template is intentionally blank and contains no patient information.

Supplementary Table S3: Complete standardized prompt template applied identically to all models and cases

Prompt component	Fixed content
(1)	Role instruction: "You are an AI decision-support system assisting an interdisciplinary medical board composed of specialists in forensic medicine, psychiatry, and neurology. Your task is to review the following standardized medico-legal case and provide an AI-assisted medical recommendation regarding legal capacity according to the Turkish Civil Code (TCC)."
(2)	Case (standardized vignette): "A 72-year-old woman was referred by the Civil Court of Peace for legal capacity assessment. She had a five-year history of Alzheimer's disease and hypertension and was receiving donepezil (10 mg/day), memantine (20 mg/day), and quetiapine (25 mg/day). The standardized vignette included the findings of independent forensic medicine, neurology, and psychiatry examinations, together with the patient's medical history, current medications, cognitive assessment (MMSE), functional assessment (ADL), and other relevant medico-legal information. Forensic medicine examination documented impaired orientation, difficulty comprehending the interview, and difficulty providing a consistent personal and medical history; neurological evaluation documented progressive cognitive decline; and psychiatric evaluation reported impaired insight and judgment. Her MMSE score was 17/30, and her ADL score was 45/100."
(3)	Legal provisions: the full statutory text of TCC Articles 405, 406, 408, and 429, exactly as reproduced in Section 2.5 of the main text, presented in fixed order.
(4)	Directive question: "Based on the standardized medico-legal case and the provisions of Articles 405, 406, 408, and 429 presented above, determine the most appropriate medical opinion regarding legal capacity. Select only one of the following categories: Article 405, Article 406, Article 408, Article 429, or No guardianship. Provide a concise justification for your selection."

Supplementary Table S4: Access details and configuration of the evaluated LLM systems.

**Item**	**ChatGPT-5.2**	**Gemini 3 Pro**	**Claude 4.5 Sonnet**
Model name displayed in the interface	ChatGPT (GPT-5.2)	Gemini (3 Pro)	Claude (Sonnet 4.5)
Developer	OpenAI	Google DeepMind	Anthropic
Access route	Public web interface (chatgpt.com)	Public web interface (gemini.google.com)	Public web interface (claude.ai)
Subscription / access type	Paid individual subscription	Paid individual subscription	Paid individual subscription
Initial evaluation dates	December 22, 2025 – January 4, 2026	December 22, 2025 – January 4, 2026	December 22, 2025 – January 4, 2026
Retest evaluation dates	January 26 – February 8, 2026	January 26 – February 8, 2026	January 26 – February 8, 2026
Model version stability	Interface identifier verified at every session; unchanged across both evaluation windows, no version transition observed	Interface identifier verified at every session; unchanged across both evaluation windows, no version transition observed	Interface identifier verified at every session; unchanged across both evaluation windows, no version transition observed
Response mode	Default settings; no custom instructions, tools, plug-ins, or retrieval augmentation	Default settings; no custom instructions, tools, plug-ins, or retrieval augmentation	Default settings; no custom instructions, tools, plug-ins, or retrieval augmentation
New isolated chat per case	Yes, for every case in both evaluation rounds	Yes, for every case in both evaluation rounds	Yes, for every case in both evaluation rounds
Conversation history / memory	Disabled; no history carried over between cases	Disabled; no history carried over between cases	Disabled; no history carried over between cases
Regeneration policy	Responses were not regenerated; the first complete response was recorded. On technical failure, refusal, or incomplete response, the case was resubmitted once in a new isolated session	Identical policy, applied uniformly	Identical policy, applied uniformly
Data-protection settings	Chat history and model-training options disabled where available in the interface	Chat history and model-training options disabled where available in the interface	Chat history and model-training options disabled where available in the interface

Supplementary Table S5: Output coding rules (Part A) and explanation-quality rubric (Part B).

Part A. Rules for mapping raw model outputs to the five predefined outcome categories (applied independently by two blinded forensic medicine specialists; disagreements adjudicated by a third specialist).

Rule	Description
1	If the output explicitly stated a single TCC article (405, 406, 408, or 429), it was coded to that category.
2	If the output explicitly recommended no guardianship or preservation of full legal capacity, it was coded as "no guardianship".
3	If more than one article was mentioned, the article presented as the primary or final recommendation was coded.
4	Outputs that were ambiguous, conditional without a definitive selection, or constituted a refusal were referred for dual independent coding and third-specialist adjudication.
5	Model identity was concealed from both raters throughout coding.

Part B. Pre-specified explanation-quality rubric (total score 0–6; adequacy threshold ≥4).

Domain	0 points	1 point	2 points
Alignment with the applicable TCC article	No reference to legal criteria, or incorrect article invoked	Partial or implicit alignment with the applicable article	Explicit and correct alignment of clinical findings with the applicable article
Consideration of the least restrictive alternative	Less restrictive alternatives not considered	Alternatives mentioned but not reasoned	Alternatives explicitly considered and reasoned against or for
Acknowledgment of uncertainty or missing information	No acknowledgment of uncertainty or missing data	Generic acknowledgment without specifics	Explicit identification of missing or uncertain clinical information relevant to the decision

Supplementary Table S6: Confusion matrices for each LLM versus IMBD recommendations at first-round zero-shot evaluation (rows: IMBD reference category; columns: model prediction; n = 234).

ChatGPT-5.2

**IMBD reference \ Model prediction**	**Art. 405**	**Art. 406**	**Art. 408**	**Art. 429**	**No guardianship**
Art. 405 (n=154)	150	0	4	0	0
Art. 406 (n=1)	0	1	0	0	0
Art. 408 (n=27)	19	0	8	0	0
Art. 429 (n=18)	11	0	0	3	4
No guardianship (n=34)	0	0	0	3	31

Gemini 3 Pro

**IMBD reference \ Model prediction**	**Art. 405**	**Art. 406**	**Art. 408**	**Art. 429**	**No guardianship**
Art. 405 (n=154)	147	0	7	0	0
Art. 406 (n=1)	0	1	0	0	0
Art. 408 (n=27)	19	0	8	0	0
Art. 429 (n=18)	2	0	0	14	2
No guardianship (n=34)	0	0	0	2	32

Claude 4.5 Sonnet

**IMBD reference \ Model prediction**	**Art. 405**	**Art. 406**	**Art. 408**	**Art. 429**	**No guardianship**
Art. 405 (n=154)	143	0	11	0	0
Art. 406 (n=1)	0	1	0	0	0
Art. 408 (n=27)	21	0	6	0	0
Art. 429 (n=18)	11	0	0	3	4
No guardianship (n=34)	0	0	0	1	33

Note: Diagonal counts correspond to the class-specific sensitivities reported in Table 2 of the main text, and each row sums to its class total. Misclassifications cluster at the Article 405–Article 408 boundary (81 of 121 pooled errors across the three models, 66.9%), with the remaining errors involving predominantly Article 429, consistent with the error pattern reported in Section 3.3.

Supplementary Table S7: Statistical comparison of classification errors across LLMs (McNemar and Friedman tests).

**Comparison**	**McNemar χ^2 (df = 1)**	**p**	**Holm-adjusted p**
ChatGPT-5.2 vs Gemini 3 Pro	2.06	0.15	0.30
ChatGPT-5.2 vs Claude 4.5 Sonnet	1.44	0.23	0.30
Gemini 3 Pro vs Claude 4.5 Sonnet	0.70	0.40	0.40
Friedman test (df = 2)	2.19	0.33	—

No pairwise difference remains significant after Holm correction; the three-way Friedman test likewise shows no LLM winner. "—" indicates not applicable (the Holm procedure applies to the pairwise McNemar comparisons only).

Supplementary Table S8: Final calibration metrics following post hoc isotonic recalibration and decision-curve analysis for the binary classification tasks (TCC Articles 405, 408, and 429).

Classification Task	Metric	ChatGPT-5.2	Gemini 3 Pro	Claude 4.5 Sonnet
TCC Article 405 vs. rest	Brier score	0.086	0.092	0.098
TCC Article 405 vs. rest	Calibration intercept	–0.02	–0.05	–0.10
TCC Article 405 vs. rest	Calibration slope	1.05	1.12	1.25
TCC Article 405 vs. rest	Net-benefit range†	0.25–0.70	0.40–0.70	0.45–0.65
TCC Article 408 vs. rest	Brier score	0.156	0.162	0.168
TCC Article 408 vs. rest	Calibration intercept	–0.04	–0.07	–0.09
TCC Article 408 vs. rest	Calibration slope	0.98	1.08	1.20
TCC Article 408 vs. rest	Net-benefit range†	0.35–0.60	0.30–0.65	None‡
TCC Article 429 vs. rest	Brier score	0.141	0.128	0.152
TCC Article 429 vs. rest	Calibration intercept	–0.03	0.00	–0.11
TCC Article 429 vs. rest	Calibration slope	1.06	1.01	1.27
TCC Article 429 vs. rest	Net-benefit range†	None‡	0.30–0.65	None‡

†Probability thresholds at which net benefit (decision-curve analysis) exceeded both the "treat-all" and "treat-none" strategies. ‡"None" indicates no positive net benefit within the pre-specified 0.10–0.80 threshold range. Calibration metrics shown are the final estimates obtained after post hoc isotonic recalibration.

Note: Because the LLMs produced only categorical recommendations, the predicted probabilities underlying these calibration and decision-curve analyses were obtained from logistic regression models fitted to the study data, in which the binary LLM recommendation for each task served as the predictor (see Section 2.8 of the main text). The values shown therefore describe the calibration properties of these secondary statistical models after post hoc isotonic recalibration, not probabilities generated directly by the LLMs.

Supplementary Table S9: Representative anonymized model outputs. Case details are condensed and fully anonymized; outputs are shown as recorded, lightly edited only for anonymization and length.

Example	Case type and condensed vignette	Model	Recorded output (recommendation and rationale)
1	Concordant Article 405 case (illustrative case of Section 2.5): 72-year-old woman, five-year history of Alzheimer's disease; MMSE 17/30, ADL 45/100; impaired orientation, insight, and judgment. IMBD: Article 405.	ChatGPT-5.2	"Recommendation: Article 405. Justification: The documented five-year history of Alzheimer's disease with progressive cognitive decline (MMSE 17/30), marked functional dependence (ADL 45/100), impaired orientation, and impaired insight and judgment indicate that the person is unable to manage her own affairs and requires continuous assistance for protection and care, satisfying the criteria of Article 405. Less restrictive measures (Article 429 or no guardianship) would not provide sufficient protection given the global cognitive and functional impairment."
2	Article 408 case misclassified as Article 405 (predominant error pattern): 81-year-old man, stroke sequelae with right-sided weakness and serious chronic illness; MMSE 24/30, ADL 55/100; partially preserved judgment documented on psychiatric examination. IMBD: Article 408.	Claude 4.5 Sonnet	"Recommendation: Article 405. Justification: The combination of advanced age, serious chronic illness, and reduced functional capacity (ADL 55/100) indicates difficulty in managing his own affairs and a need for continuous assistance and protection; therefore guardianship under Article 405 is the most appropriate measure." (Coded error: the output emphasized diagnosis and functional dependence without weighing the partially preserved decision-making capacity or the request-based, proportionate protective character of Article 408.)
3	Concordant no-guardianship case: 45-year-old man, anxiety disorder under outpatient treatment; MMSE 29/30, ADL 100/100; no impairment of judgment, orientation, or daily	Gemini 3 Pro	"Recommendation: No guardianship. Justification: The findings do not demonstrate any impairment of cognition, judgment, or the ability to manage personal and financial affairs. An

	functioning on any examination. IMBD: no guardianship.		anxiety disorder under regular outpatient treatment, with intact cognitive testing (MMSE 29/30) and full functional independence (ADL 100/100), does not satisfy the criteria of Articles 405, 406, 408, or 429, and legal capacity should be preserved."
4	Safety-flagged output (Article 405 recommended without clear cognitive or functional justification): 58-year-old woman, schizophrenia in documented remission under depot antipsychotic treatment; MMSE 27/30, ADL 90/100; no current psychotic findings. IMBD: Article 429.	ChatGPT-5.2	"Recommendation: Article 405. Justification: The presence of a chronic psychotic disorder indicates that the person may endanger her own interests and requires guardianship for protection." (Flag reason: recommendation based on the diagnostic label alone, without cognitive or functional justification and without consideration of less restrictive alternatives; the case underwent mandatory senior review in accordance with Section 2.10.)

Note: Examples were selected to illustrate a concordant Article 405 recommendation, the predominant Article 408 to Article 405 error pattern, a concordant no-guardianship recommendation, and a safety-flagged output. All identifying details have been removed.

Supplementary Table S10: Cross-jurisdictional comparison of guardianship frameworks under the Turkish Civil Code (TCC), the UK Mental Capacity Act 2005, the German Civil Code (BGB), and the Swiss Civil Code (ZGB).

TCC Provision	Scope	UK–MCA 2005	Germany–BGB	Switzerland–ZGB	Rights Protection
Art. 405 Full Guardianship (mental illness or mental deficiency)	Lacks capacity for essential civil acts; guardian acts in all legal & financial domains	s.16 deputyship (general authority); least-restrictive option; COP3 capacity form	§1814 Betreuer appointment; §1825 consent-reserve (Einwilligungsvorbehalt)	Art. 398 Umfassende Beistandschaft (last resort)	Core personal rights preserved; intervention only when necessary
Art. 408 Guardianship upon the Person's Own Request (old age, disability, inexperience, or serious illness)	Established upon the adult's own request where inability to manage his or her own affairs is proven; protective and proportionate in scope	Limited s.16 deputyship; domain-specific authority	§1814 limited guardian; §1825(2) domain consent	Art. 394–395 Mitwirkungs-/Vertretungsbeistandschaft	Proportionality; targeted support; promotes autonomy
Art. 429 Advisor / Consent-Linked Support	Capacity retained; advisor consent required for specific high-risk acts	Narrow judicial deputyship; best-interests test	§1825 consent reserve without loss of autonomy	Art. 395 supported decision-making with consent	Individual retains control; limited intervention
No Guardianship (Full Capacity)	Full capacity; no intervention; support encouraged if needed	No deputyship; informal support possible	§1814(3) intervention only if required; subsidiarity	General capacity; intervention only by formal order	Autonomy and self-determination prioritized
Art. 406 Prodigality, Addiction, or Harmful Lifestyle	Financial vulnerability due to prodigality, addiction, or harmful lifestyle placing self or family at risk	s.16 deputyship limited to finances	§1814 limited financial scope; §1825 consent for financial acts	Art. 394–395 financial-domain support	Property protection; lifestyle autonomy preserved

Note: This table provides an approximate conceptual comparison only. Guardianship, supported decision-making, legal capacity restrictions, and advisor systems are not fully equivalent or legally interchangeable across the Turkish, UK, German, and Swiss legal systems, and the correspondences shown should not be interpreted as exact legal equivalents. The English descriptions of the TCC categories are functional summaries prepared by the authors and do not constitute official translations.
